# Trends in Plant-Based Diets and the Associated Health Characteristics among Canadians

**DOI:** 10.3390/nu16162628

**Published:** 2024-08-09

**Authors:** Zoe Lillian Longworth, Razieh Mohammadkhani, Michael Szafron, Ginny Lane, Hassan Vatanparast

**Affiliations:** 1 College of Pharmacy and Nutrition, University of Saskatchewan, Saskatoon, SK S7N 5E5, Canada; zoe.longworth@usask.ca; 2 School of Public Health, University of Saskatchewan, Saskatoon, SK S7N 5E5, Canada; xsz274@mail.usask.ca (R.M.); michael.szafron@usask.ca (M.S.); 3 Family and Consumer Sciences, College of Agricultural & Life Sciences, University of Idaho, Moscow, ID 83844, USA

**Keywords:** plant-based diet, dietary pattern, diet quality, Nutrient-Rich Food index, chronic conditions, public health, Canada

## Abstract

In Canada, unhealthy dietary patterns comprise diets with poor nutrient density and are associated with chronic conditions. Plant-based diets have gained popularity due to their ability to provide a nutritionally adequate healthy diet. This study aims to compare sociodemographic, socioeconomic, and health characteristics, and diet quality between Canadian adults following plant-based and omnivore diets as well as assess the extent to which key nutrient intakes are of public health concern among Canadians following plant-based diets. The study used nationally representative nutritional data from the 2015 Canadian Community Health Survey and descriptive statistics were computed. The analysis determined that Canadians following strict plant-based diets (1% of total population) were significantly more likely to be an immigrant to Canada, less likely to meet national physical activity guidelines, and less likely to be overweight, compared to Canadians following omnivore diets. Compared to omnivore diets, plant-based diets were nutritionally superior according to the Nutrient-Rich Food index. Continued knowledge translation on what comprises healthy plant-based diets, public guidance on the intersection between diet and health, and the completion of prospective cohort studies are needed. To conclude, the research suggests well-planned plant-based diets, in comparison to omnivore diets, offer a nutrient-dense diet.

## 1. Introduction

Food systems and individual dietary patterns have the potential to nurture human health and support environmental sustainability; however, this is often not attained [1]. Unhealthy diets pose a great risk towards morbidity and mortality in humans—a risk greater than the combined impact of unsafe sex and the use of alcohol, drugs, and tobacco [1]. Environmentally, unsustainable agriculture and food production can negatively impact freshwater use, land use, and greenhouse gas emissions, and negatively impact coastal waters and wetlands through a process called eutrophication, where plant and algae growth increases by mediating the factors needed in the natural photosynthesis process, and, as a result, have a myriad of detrimental impacts on the ecosystem [2,3]. Therefore, what is the solution to healthier dietary patterns and sustainable food production? Plant-based diets have entered the spotlight as a dietary pattern that may help support both human and environmental health, or planetary health [2,3,4]. Through an environmental lens, the adoption of plant-based diets has the potential to significantly reduce agricultural land use by 76%, freshwater use by 21% (blue water [water in rivers, lakes, and glaciers] and groundwater), eutrophication by 49%, and greenhouse gas emissions by 49% [2]. Additionally, raising animals for human consumption is responsible for 67% of deforestation, threatening our ecosystems and biodiversity [2]. Through a human health lens, the adoption of well-planned plant-based diets has the potential to improve cholesterol levels, glycemic control, blood pressure, and weight management while reducing the incidence of cardiovascular diseases, type 2 diabetes (T2D), and obesity [2,3,4]. However, in the context of human health, the distinction of a well-planned or a ‘healthy’ plant-based diet should be made, as opposed to a plant-based diet high in sugar, salt, saturated or trans-fat, or highly processed foods. As with any dietary regimen, there is the possibility to over- or under-consume essential nutrients or nutrients to limit (i.e., saturated fats, sugar, and sodium) [2,3,4].

In 2019, both the EAT-Lancet Commission and Health Canada independently defined new optimal diets, both of which emphasized plant-based foods and proteins [5,6]. The EAT-Lancet Commission defined a diet with the intent to support optimal planetary health, which consists largely of a diversity of plant-based foods, low amounts of animal sourced foods, primarily unsaturated fats instead of saturated, and limited amounts of refined grains, processed foods, and added sugars [5]. More specifically, the planetary health diet is made up of 50% vegetables and fruits (by volume) or about 300–900 g/day (78 kcal/day vegetables, 126 kcal/day fruits) including other major contributing food groups of whole grains (232 g/day or 811 kcal/day), plant-based proteins (0–175 g/day or 575 kcal/day), and unsaturated oils (20–80 g/day or 354 kcal/day) [5]. Similarly, the 2019 Canadian Food Guide dedicates half of the plate to vegetables and fruits, with whole grains and protein foods each making up one-quarter of the plate [6]. Although much less quantifiable, the 2019 Canadian Food Guide emphasizes plant-based proteins and recommends Canadians choose protein foods from plants every day [6]. These two plant-focused dietary guidelines highlight a Canadian and global movement towards plant-based eating for both human and environmental health.

In the Canadian context, our population is experiencing epidemic proportions of chronic conditions, with significant impacts on population health, the economy, and our society [4]. Based on 2018 Canadian Community Health Survey (CCHS) data, more than half of our population was classified as overweight or obese (36.3% overweight, and 26.8% obese) [7]. Evidently, carrying excess weight predisposes individuals to increased health risks, including the increased risk of several chronic conditions such as T2D, hypertension, cardiovascular diseases, and metabolic syndrome [8]. The number of Canadians impacted by chronic conditions will continue to rise due to our aging population, sedentary lifestyles, and unhealthy dietary patterns [4]. Fortunately, our dietary patterns are a modifiable risk factor that influences our health and wellness. Dietary patterns and specific foods have been linked to several chronic conditions such as cardiovascular diseases, T2D, hypertension, metabolic syndrome, some cancers, and, as mentioned above, obesity [4,9,10,11]. Red and processed meats have been classified as ‘probably carcinogenic to humans’ and ‘carcinogenic to humans’, respectively, since 2015 by the International Agency for Research on Cancer, and new evidence continues to support these classifications [12]. However, to have a comprehensive understanding on diet and chronic conditions, researchers have recently focused more on overall dietary patterns, rather than individual foods. Several studies have investigated the role of plant-based dietary patterns on chronic condition outcomes and have identified the health-promoting characteristics of these dietary patterns. An analysis of the second American Adventist Health Study found an association between plant-based dietary patterns and reduced odds of developing T2D, hypertension, high cholesterol, and obesity [13,14]. Further, a Canadian randomized controlled trial investigating the differences between types of plant-based diets found that consuming a low-carbohydrate vegan diet over 6 months resulted in reductions in body weight and in low-density lipoprotein (LDL) cholesterol, compared to those consuming a high-carbohydrate lacto-ovo vegetarian diet [15]. Investigating overall dietary patterns provides a more practical and realistic population health perspective of how our diets relate to chronic conditions; however, specific nutrients of public health concern still need to be acknowledged and investigated. For instance, there is a well-established relationship between vitamin D and calcium on bone health and it is imperative to ensure that one’s diet has a sufficient intake of these important vitamins and minerals [11]. Additionally, within a population health lens, understanding the distribution of social determinants of health (e.g., household income, food security status, education level, immigration status, etc.) among a population helps provide a comprehensive understanding of the additional variables at play that also contribute to one’s health status [16]. To date, there is a lack of relevant Canadian population-based research assessing the impact of plant-based eating on diet quality, nutrients of public health concern, and the prevalence of chronic conditions, as well as the distribution of plant-based eating across sociodemographic and socioeconomic factors.

Therefore, it is imperative that we investigate the population impact of following plant-based and omnivore dietary patterns on diet quality and nutrient intake metrics among Canadians. The adoption of healthy plant-based diets may address the significant need to improve the health of Canadians [4]. Thus, we set out to examine the trends of Canadians practicing plant-based dietary patterns and the resulting impact on nutrient density. Our specific objectives were: (i) to compare the sociodemographic, socioeconomic, and health characteristics between Canadian adults following plant-based diets to those following an omnivore diet; (ii) to compare diet quality between Canadian adults following plant-based diets to those following an omnivore diet; and (iii) to assess the extent to which key nutrient intakes are of public health concern among Canadians following plant-based diets.

## 2. Materials and Methods

### 2.1. Survey Design

This study is based on nutritional data from the 2015 CCHS, a nationally representative cross-sectional survey. The CCHS is a national cross-sectional survey that relied on voluntary responses from the Canadian population and executed a three-stage stratified data collection process to allow for inferences at a national level [17]. The CCHS 2015 was selected as it contains the most recent focused surveys on nutrition, the CCHS-Nutrition, which include 24 h dietary recalls.

### 2.2. Study Population

The CCHS study population represented the Canadian population aged 1 year and older living in 1 of the 10 provinces, while excluding individuals living on reserves or other Indigenous settlements, full-time members of the Canadian Forces, or the institutionalized population [17]. At the national level, Statistics Canada desired a sample size of 24,000 respondents; therefore, after accounting for non-response and out-of-scope dwellings, a total of 37,694 households were selected, and the overall response rate at the national level was estimated to be 62% [17].

**Figure 1:**
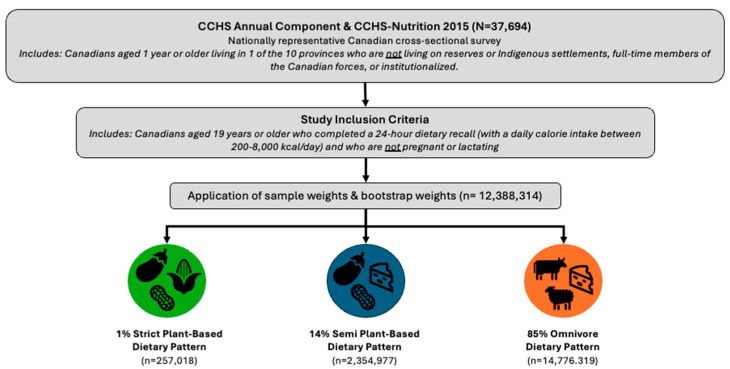
Overview of study inclusion/exclusion criteria and sample sizes.

A subset of the CCHS-Nutrition study population was used for the purposes of this study and is referred to as the analytical sample. The analytical sample excluded individuals if they were less than 19 years of age, pregnant, lactating, did not report any food intake in the 24 h dietary recall, or reported a daily calorie intake outside the range of 200–8000 kilocalories. These criteria were selected to focus on Canada’s adult population and minimize potential confounders (i.e., those who are pregnant or lactating) and minimize outliers (i.e., those who are consuming less than 200 kcal/day or more than 8000 kcal/day). With consideration of sample weights, bootstrap weights, and the exclusion criteria, the analytical sample based on the weighted sample represent 17,388,314 Canadian adults living in 10 provinces (Figure 1).

### 2.3. Variable Definitions

The CCHS Annual Component and the focused CCHS-Nutrition module collected information on a variety of population health measures, such as household food security, disease and health conditions, lifestyle and social conditions, healthcare services, mental health, and wellbeing [18]. For this study, variables related to sociodemographics, socioeconomics, nutrient intakes, and status of several chronic conditions were accessed, such as T2D, hypertension, heart disease, cancer, or osteoporosis. Overweight and obesity status was classified using body mass index (BMI), such that individuals with a BMI of >18.5 were classified as underweight, individuals with a BMI of 18.5–24.9 were normal weight, individuals with a BMI of 25.0–29.9 were overweight, and individuals with a BMI ≥ 30 were classified as obese. Physical activity was assessed across leisure, work, housework, and transportation where all respondents reported the number of times and the duration of moderate or vigorous physical activity in the previous seven days [17]. These data were used alongside the national physical activity recommendation to classify individuals as to whether or not they participated in 150 min, or more, of moderate or vigorous physical activity per week [17]. Household food security status was assessed using the 18-item Household Food Security Survey Module. For the purposes of this study, household food security was reported as two levels: food-secure and food-insecure (includes both moderate and severe food insecurity) [17,19]. Nutritional supplementation was assessed by both the type and volume of supplements consumed and the frequency of supplement consumption in the previous month [17]. CCHS respondents were asked to report on supplement brand, ingredients, concentration, and, when available, the Natural Product Number [17]. For the purposes of this study, nutritional supplementation was classified into respondents who reported consumption in the past month and respondents who did not. Furthermore, three variables were derived to help guide the research process—the priori dietary pattern variable, the Nutrient-Rich Food (NRF) index variable, and a total chronic conditions variable.

The dietary pattern variable was derived using participants’ 24 h dietary recall data that included food type, amount, eating occasions and locations, and supplement intake. CCHS-Nutrition respondents completed one 24 h dietary recall, and a random subset of approximately 35% of the sample was invited for a second 24 h dietary recall. In most cases, the first 24 h dietary recall was completed in-person; however, some were completed over the phone, and all recalls were completed using the Automated Multiple-Pass Method [17]. For the purposes of this study, only the first 24 h dietary recall was used in analysis. One 24 h dietary recall provides sufficient dietary data to provide population level estimates of mean intake and is not statistically different than the mean of two 24 h dietary recalls. The derived dietary pattern variable stratified respondents into three unique diet groups—omnivore, semi-plant-based (lacto-ovo vegetarian), or strict plant-based (vegan), following the commonly accepted dietary patterns [20]. A respondent’s diet was classified as omnivore if they reported consumption of one or more servings of any animal products (i.e., beef, pork, veal, lamb, game meat, organ meat, processed meat, poultry, fish, and shellfish) during the first 24 h dietary recall. A respondent’s diet was classified as semi-plant-based if they reported no consumption, or zero servings, of animal products but reported consumption of at least one serving of animal by-products (i.e., milk, cheese, yogurt, and eggs) and at least one serving of fruits/vegetables during the first 24 h dietary recall. A respondent’s diet was classified as strict plant-based if they reported no consumption, or zero servings, of animal products or by-products but reported consumption of at least one serving of fruits/vegetables during the first 24 h dietary recall.

The NRF index variable was computed from an adapted version of the validated NRF 9.3 index, which is a formal metric used to assess overall diet quality, with a high score associated with positive health outcomes [21,22]. The NRF index variable was derived by subtracting the sum percentage of the daily values of nine nutrients to encourage (protein, fibre, vitamin A, vitamin C, vitamin D, calcium, iron, magnesium, and potassium) with the sum percentage of the maximum recommended values for three nutrients to limit (saturated fat, total sugar, and sodium). Higher NRF index scores (maximum 900) indicate a nutrient-rich diet [21,22]. NRF index tertiles were derived and categorized as low score (tertile 1, NRF ≤ 465.97), medium score (tertile 2, NRF > 465.97 and < 578.42), or high score (tertile 3, NRF ≥ 578.42). The NRF tertiles represent three equal groups making up the entire sample population; however, when the sample population is stratified into dietary patterns, the distribution of the population in each tertile is not equal.

The total chronic conditions variable was developed to complete a broader analysis on the relationship between dietary patterns and the occurrence of at least one of the following chronic conditions—T2D, hypertension, heart disease, cancer, or osteoporosis. The total chronic condition variable was defined by whether the respondent either answered ‘yes’ to at least one of the indicated chronic condition status questions or answered ‘no’ to all of the indicated chronic conditions.

### 2.4. Statistical Analyses

Statistical analyses were completed using SAS (Version 9.4, SAS Institute, Toronto, ON, Canada) software at the Saskatchewan Research Data Centre located at the University of Saskatchewan. To extrapolate results to the national level and produce population level estimates, survey weights and bootstrap weights provided by Statistics Canada were applied to all analyses [18]. A stepwise process was used throughout analysis such that (i) data was cleaned and processed by merging datasets by respondent ID, adding bootstrap and sample weights, developing of variable levels, derivation of variables, and incorporation of exclusion criteria, followed by (ii) computation of descriptive statistics at both the sample level and with recommended bootstrap and sample weights. For descriptive statistics, all categorical variables were computed as weighted frequencies whereas continuous variables were computed as weighted means. For all variables, statistics were reported with the standard error and 95% confidence interval per each dietary pattern classification. Statistical significance was determined via the confidence interval approach, recommended by Statistics Canada for analyses on CCHS data, such that, if the 95% confidence intervals overlapped, the parameters were statistically equivalent, or, alternatively, if the 95% confidence intervals did not overlap, the parameters were determined to be statistically different.

### 2.5. Ethical Considerations

Ethical review and approval were waived for this study as research pertained to secondary analysis of the CCHS data. In Canada, ethical review and approval are waived when the research conducted relies exclusively on information that is publicly available through a mechanism set out by legislation or regulation and that is protected by law, or in the public domain, and the individuals to whom the information refers have no reasonable expectation of privacy.

## 3. Results

**Figure 2:**
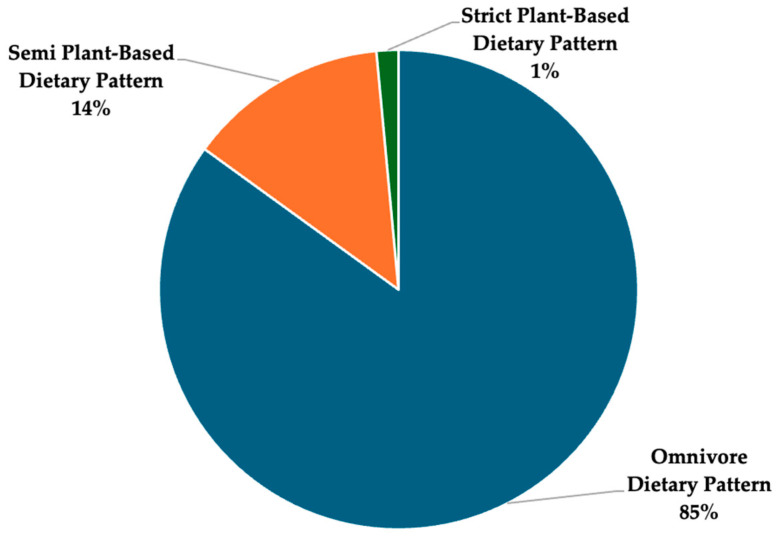
Distribution of dietary patterns among Canadians.

This study included a weighted sample size of 17,388,314 Canadians and explored the population level trends of dietary patterns (omnivore, semi-plant-based, and strict plant-based) associated with the sociodemographic/socioeconomic factors, health characteristics, diet quality, and nutrients of public health concern. Approximately, 84.98% of the national population follows an omnivore diet, 13.54% follows a semi-plant-based diet, and 1.48% follows a strict plant-based diet (Figure 2).

### 3.1. Dietary Patterns in Canada

Table 1 presents the sociodemographic, socioeconomic, and health characteristics of adults following an omnivore diet, a semi-plant-based diet, and a strict plant-based diet. There were significant differences between the dietary pattern groups associated with sex, immigration status, physical activity, and BMI. For instance, Canadians following a semi-plant-based diet were significantly less likely to be male compared to those following an omnivore diet. Further, compared to Canadians following an omnivore diet, those following a strict plant-based diet were significantly more likely to be an immigrant to Canada, less likely to meet national physical activity recommendations, and less likely to be overweight. There were no significant findings related to chronic condition status, aside from BMI, across the three dietary patterns. However, Canadians following a strict plant-based diet had a higher, yet insignificant, prevalence of total chronic conditions compared to semi-plant-based or omnivore Canadians.

### 3.2. Diet Quality of Canadian Dietary Patterns

Table 2 outlines the distribution of the NRF index scores in each dietary pattern, as well as the mean NRF index score for each dietary pattern. Canadians following a semi-plant-based diet were significantly less likely to be classified in the low NRF index score group (lower nutrient density) and significantly more likely to be classified in the high NRF index score group (higher nutrient density) than their omnivore counterparts. Moreover, Canadians following a semi-plant-based diet had a significantly higher mean NRF index score, representing a more nutrient-dense diet, compared to their omnivore counterparts.

### 3.3. Key Nutrient Intakes in Canadian Dietary Patterns

Key nutrients, including nutrients of public health concern, are summarized in Table 3 and Table 4 along with their associated standard error and 95% confidence intervals for each dietary pattern. Numerous mean nutrient intakes were found to be statistically different between dietary groups; however, the most notable differences were found between Canadians consuming an omnivore diet and Canadians consuming either a semi- or strict plant-based diet. Regarding micronutrients, those practicing a strict plant-based diet had significantly lower intakes of vitamin A, vitamin B, vitamin B2, vitamin D, calcium, cholesterol, mono-unsaturated fatty acids (MUFAs), saturated fatty acids, iron, niacin, phosphorus, riboflavin, sodium, thiamin, and zinc, compared to Canadians practicing an omnivore diet. Micronutrient intakes associated with the semi-plant-based diet tended to fall somewhere between the mean nutrient intakes of the omnivore dietary pattern and the strict plant-based dietary pattern. Nevertheless, Canadians following a semi-plant-based diet had significantly higher mean intakes of folate and dietary fibre, compared to Canadians practicing an omnivore diet. Regarding macronutrients, Canadians practicing either form of the plant-based diet (semi- or strict plant-based) had significantly lower mean intakes of total fat, protein, and energy, compared to their omnivore counterparts.

## 4. Discussion

The results presented provide a novel overview on the distribution of omnivore and plant-based diets in Canada and the trends related to sociodemographics, health characteristics, diet quality, and potential nutrients of concern. The analysis determined that Canadians consuming some form of plant-based dietary pattern had certain sociodemographic, socioeconomic, and health trends; for instance, sex, immigration status, physical activity, and body weight were found to be significant. Furthermore, Canadians consuming a semi-plant-based diet were found to have the highest NRF index score and, upon individual nutrient analysis, were found to have sufficient nutrient intakes of common public health nutrients of concern.

### 4.1. Sociodemographic, Socioeconomic, and Health Trends in Plant-Based Eating

Approximately 15% of the Canadian adult population practice some form of plant-based eating. The present analysis found that Canadians consuming either plant-based diet (semi-plant-based or strict plant-based) were significantly more likely to be female, an immigrant to Canada, not meet national physical activity recommendations, and not be overweight, compared to their omnivore counterparts. Studies have commonly reported that females are more likely to report the consumption of ‘healthy’ dietary patterns or to practice plant-based dietary patterns compared to males [15,23,24,25,26,27,28]. Similarly, other research often finds individuals practicing plant-based diets to have healthy weights, or fall within normal BMI ranges [23,24,25,26]. However, a comparable Canadian study which also analyzed the CCHS-N 2015 but included a wider age range of participants (greater than 2 years of age) found that only 5% of the population reported following some form of plant-based diet [26]. The discrepancy between Valdes et al.’s study and the present study is likely due to the inclusion of young children. A recent 2021 American survey reported that only about 5% of children aged 5 to 17 in the U.S. describe their dietary pattern as vegetarian, or plant-based [29]. Plant-based eating is generally common among younger adult populations, but this trend does not typically include children [29].

Compared to Canadian-born individuals, immigrants to Canada were significantly more likely to practice a strict plant-based diet and less likely to practice an omnivore diet; however, this trend did not remain significant when controlling for the length of time in Canada. Reasons for this observed trend may include the focus on plant-based eating in many non-Western cultures or the higher prevalence of food insecurity among recent immigrants [28,30]. Many cultures, religious groups, and geographical regions have deep connections to plant-based eating, such as Hindus, Buddhists, and Jains, as well as populations living in specific regions such as Okinawa, Japan. Religious-inspired vegetarianism, or plant-based eating, involves limiting one’s dietary intake with the aim of attaining spiritual enlightenment or self-fulfillment, or as a mark of respect for the regulations set by one’s religious community [31,32]. For example, due to the dietary guidelines outlined in the Hindu faith, which permit the consumption of only plant-based foods and dairy products, Hindus have traditionally been recognized as lacto-vegetarians [33]. Furthermore, the geo-spatial influence of a community, or cultural group’s region, can impact dietary patterns such as in the local population of Okinawa, Japan, who traditionally adhere to a predominantly plant-based dietary pattern characterized by a high consumption of phytochemicals and antioxidants [34]. Their plant-based diet is rich in sweet potatoes, green leafy vegetables, and soy products with minimal amounts of fat (less than 6%) [34]. Okinawa, Japan has been referred to as one of the world’s ‘Blue Zones’, which reflects a demographic and geographic area with a high population longevity, exemplifying the role of healthful plant-based eating on one’s health and wellbeing [34].

The second possible rationale for the higher prevalence of immigrants to Canada practicing strict plant-based diets could be due to social determinants of health, namely, food security status. Food insecurity can disproportionately affect newcomers to Canada and plant-based eating, specifically traditional plant-based proteins (i.e., nuts, seeds, and legumes), can offer inexpensive alternatives to omnivore diets and animal-based products [30,35]. For instance, a recent modeling study found that plant-based diets which focused on legumes and whole grains in place of animal products represented the most affordable dietary pattern [35]. Springmann et al. (2021) [35] found that, in high- and upper-middle-income countries, meat and meat products accounted for the greatest proportion of food costs, approximately 32–34%. At a regional level, many community-based organizations promote plant-based proteins as inexpensive alternatives to animal-based proteins. For example, a six-week cooking program involving the provision of plant-based food hampers and cooking classes using plant-based recipes resulted in participants cooking more plant-based meals, having reduced BMIs, and reduced food insecurity from baseline [36]. At an international level, transitioning from animal-based diets to plant-based diets can support achieving global food security and environmental sustainability goals, primarily by shifting major crops away from livestock feed and biofuels to crops for human consumption, such as soy and grain [37]. However, it should not go without mention that the cost of plant-based dietary patterns, or any dietary pattern, is highly dependent on one’s geographical location (i.e., rural, remote, and northern) and access to grocery stores or arable land. As a result, plant-based dietary patterns including nuts, seeds, and legumes may not be the most economical choice in some locations.

Our analysis revealed that 45.44% of individuals following an omnivore diet met the national physical activity recommendations, whereas individuals following a strict plant-based diets were significantly less likely to meet recommendations at 26.74% (Table 1). This observed difference between physical activity levels may be influenced by confounding factors such as sex or age. For instance, our analysis found that a significantly higher proportion of males followed an omnivore diet, compared to a semi-plant-based diet. However, although non-significant, Canadians following a strict plant-based diet were younger and more likely to be male, compared to their omnivore counterparts. Irrespective of this funding, the consumption of a nutritionally balanced plant-based diet has not been shown to have a detrimental effect on performance outcomes and may even improve aerobic capacity in athletes [38]. There is a common misconception that plant-based diets, specifically vegan or semi-plant-based diets, are low in protein or lack complete protein sources; however, this is not the case if the plant-based diet is well-balanced and protein intake is considered a priority along with other micro- and macro-nutrients [39]. Previous research has also determined that individuals who follow an omnivore diet do not exhibit any distinct benefit or drawback in terms of strength, anaerobic, or aerobic exercise performance in comparison with people who follow vegetarian or vegan diets [40]. Considering the high carbohydrate content, antioxidants, and phytochemicals present, plant-based diets can provide both health advantages and potential performance-enhancing effects for exercise [41]. Further mixed-methods research should be completed to better understand the potential association between plant-based diets and physical activity and delve into why these relationships may exist.

### 4.2. Sufficient Diet Quality in Plant-Based Eating

Our analysis found Canadians practicing the semi-plant-based diet had the highest mean NRF 9.3 index score and the largest proportion in the high NRF index score group (higher nutrient density), compared to Canadians practicing an omnivore diet. This statistically significant finding indicates that Canadians following a semi-plant-based diet have a superior diet quality compared to their omnivore counterparts. This finding is comparable to an American study, based on a cross-sectional nationally representative sample, which found American adults following a semi-plant-based diet had a more nutrient-dense diet compared to their omnivore counterparts [42]. Further, a Swedish study found that individuals following a strict plant-based diet, or vegan diet, had the highest Healthy Eating Index (HEI) score, and all plant-based diets under study had significantly higher HEI scores compared to an omnivore diet [43]. A systematic review by Parker and Vadiveloo (2019) [44] reported that vegetarians scored 4.5 to 16.4 points higher on the HEI than non-vegetarians, out of a possible 100 points. Policy documents build on these scientific findings, such as the 2019 Canadian Food Guide, which supports a transition to the increased consumption of plant-based proteins and reduced consumption of animal products compared to previous Canadian Food Guides [6]. According to a recent study by Asher, Doucet, and Luke (2021) [45], Canadian dieticians support the 2019 Canadian Food Guide, believe it was informed by evidence-based information, and have adapted to recommending plant-based foods more often in their practices. There is a consensus among the scientific literature that plant-based diets can provide a diet of high nutritional quality.

Regardless of the significant evidence supporting the transition to plant-based eating patterns, there is wide nutritional variation within various plant-based diets. Plant-based eating generally focuses on a diet that is high in fruits, vegetables, whole grains, and legumes, with the exclusion, to various degrees, of animal products. However, a plant-based diet can also include various unhealthy food options such as refined grains or sugar. Research has shown that plant-based dietary patterns that prioritize less nutritious plant foods like sugary beverages, refined grains, fried foods, and sweets are associated with increased risks of cardiovascular disease, T2D, metabolic syndrome, and overall mortality [46]. Consumers see the term ‘plant-based’ on products and assume that all of these products are automatically healthy, while plant-based ultra-processed foods have the potential to contain elevated levels of undesirable nutrients like saturated fat, sugar, and salt [47]. Therefore, plant-based diets are not a blanket term that consistently means the same thing. A deeper understanding of healthy or unhealthy plant-based diets in the Canadian context is warranted.

### 4.3. Sufficient Nutrient Intakes in Plant-Based Eating

Numerous nutrients were consumed in statistically lower amounts among Canadians practicing either of the plant-based dietary patterns, compared to those following an omnivore diet. Most notably, Canadians practicing a semi-plant-based diet had significantly lower mean intakes of vitamin B6, vitamin B12, cholesterol, niacin, zinc, and total protein, but higher mean intakes of dietary fibre and folate, compared to Canadians practicing an omnivore diet. Canadians practicing a strict plant-based diet had significantly lower mean intakes of vitamin A, vitamin B6, vitamin B12, vitamin D, calcium, cholesterol, MUFAs, SFAs, iron, niacin, phosphorus, riboflavin, sodium, thiamin, zinc, total fat, total protein, and total calories compared to Canadians practicing an omnivore diet. Some of these significant differences associated with consuming a plant-based diet can be viewed in a positive manner, such as the reduced intake of cholesterol, SFAs, and sodium, as well as higher intakes of folate and dietary fibre. However, some of these significant differences may indicate concerns, such as the reduced intake of vitamin B6, vitamin B12, niacin, zinc, vitamin D, calcium, and iron. Previous research on nutrient intakes among individuals consuming plant-based diets have found similar concerns [21,42,43,48,49,50,51,52,53]. The conventional nutrients of concern in plant-based eating seem to be generally well-agreed-upon, including sufficient vitamin B12, iron, zinc, calcium, vitamin D, and omega-3 fatty acids intakes [53]. Another common concern of plant-based nutrition is the adequate intake and quality of protein sources [43,48,51,52,54]. Animal-based proteins are often designated as ‘complete’ proteins as they contain all nine essential amino acids, whereas plant-based proteins are often ‘incomplete’ as they frequently fall short of containing all nine essential amino acids [43,48,51,52,54]. However, not all plant-based proteins fall short of the same amino acids; therefore, multiple plant-based proteins sources can be eaten together to create a ‘complete’ protein source [48,51,54]. As such, plant-based diets should be well-planned to ensure the adequate consumption of these nutrients [21,42,43,48,49,50,51,52]. A well-planned plant-based diet considers a variety of foods with an emphasis on fruits, vegetables, whole grains, and conventional plant-based proteins such as soy, beans, and nuts.

Furthermore, the present study found a significant difference in mean nutrient intakes between the semi- and strict plant-based diets, meaning that not all plant-based diets are equivalent. Canadians practicing a semi-plant-based diet had significantly higher intakes of vitamin B12, vitamin D, calcium, cholesterol, MUFAs, SFAs, niacin, phosphorus, riboflavin, sodium, zinc, total fat, total protein, and total calories compared to Canadians practicing a strict plant-based diet, indicating the consumption of animal by-products makes an important contribution to nutrient intakes. Not all plant-based diets are equivalent—there is an imperative difference between healthy and unhealthy plant-based foods. For instance, a case study by Tso and Forde (2021) [54] modelling the difference in diets focused on traditional plant-based foods (i.e., legumes, nuts, and soy) and diets focused on novel plant-based meat alternatives (i.e., prepared burgers, nuggets, and diary alternatives) found the traditional plant-based food diets met daily nutritional requirements for potential nutrients of concern (i.e., calcium, iron, zinc, and vitamin B12) and had lower amounts of SFAs, sodium, and sugar compared to the reference omnivore diet. In contrast, the diet focused on novel plant-based meat alternatives did not meet daily nutritional requirements for potential nutrients of concern (i.e., calcium, zinc, and vitamin B12) and had increased intakes of SFAs, sodium, and sugar compared to the reference diet [54]. A cross-sectional analysis on the CCHS 2015 study population found that only 14.5% of Canadians reported consuming traditional plant-based meat alternatives such as legumes, nuts, and seeds [52]. Interestingly, when the authors modelled the nutritional impact of reducing processed meat consumption by 50% and doubling traditional plant-based meat alternatives, there were statistically significance increases in the dietary intake of fibre, PUFAs, magnesium, and folate equivalents paired with significant decreases in protein, cholesterol, zinc, and vitamin B12 [52]. These findings highlight the importance of the type and quality of plant-based foods consumed, as well as emphasize that a well-planned plant-based diet can be nutritionally adequate and beneficial to health.

### 4.4. Strengths and Limitations

This analysis provides valuable information on the trends and nutritional quality of plant-based eating in Canada and fills a significant knowledge gap based on the most recent nationally representative nutrition and health survey in Canada. The use of the CCHS-Nutrition data should be considered a strength as the survey data were well-suited to this type of nutritional analysis. CCHS-Nutrition data provided nationally representative information on nutrient and food intakes as well as sociodemographic, socioeconomic, diet, health, and lifestyle factors that can be extrapolated to the Canadian population. Irrespective of the strengths, the limitations of our analysis should be acknowledged. First, dietary pattern classifications were derived based on one self-reported 24 h dietary recall, which may not accurately represent the totality of a participant’s usual diet. Dietary groups were defined at the population level based on the inclusion or exclusion of animal products and by-products, and the consumption of at least one serving of fruits and vegetables to be placed in the plant-based dietary patterns groups. As this method is population-based and not specifically focused on healthful or nutritionally balanced plant-based diets, it offers a realistic comparison between the inclusion and exclusion of animal products and by-products based on the group mean intake. Nevertheless, much of the published literature defines dietary groups in different manners, and, thus, caution must be taken when comparing research results. Second, the 95% confidence interval approach to determining statistical significance is a conservative approach and, as a result, although it increases the rigor of the analyses, it is plausible that the results may include false negatives. Third, as a result of the data distribution, small sample sizes occurred in some variables resulting in the need to combine or merge some variables (i.e., ethnicity, household income, BMI, food security, and marital status) to meet Statistics Canada reporting requirements. Special considerations were made when combining variable levels; however the variables with combined levels may not accurately represent the diversity of the variable in question. Finally, there are various limitations that should be declared due to the nature of the CCHS-Nutrition study design. Large cross-sectional studies measure all factors simultaneously, which restricts calculations of incidence and determination of temporal relationships, and introduces the potential of reverse causation. Non-response bias and recall bias could also figure prominently in cross-sectional studies; however, Statistics Canada took precautions to limit these biases and obtain a high response rate. Due to the nature of the CCHS-Nutrition study population parameters, an Indigenous population was included in the analysis, but excluded individuals living on First Nation reserves; the statistical analysis presented does not represent the entirety of the Canadian Indigenous population; however, this is minor as it includes urban Indigenous populations. Further, the data used in the analysis were gathered in 2015 and may not accurately represent the current trends of Canadian dietary patterns as the social acceptance and popularity of plant-based eating has increased within the past five years.

## 5. Conclusions

The present study based on the CCHS-Nutrition data indicates the superior diet quality of plant-based diets. In Canada, scientific evidence and social support of plant-based eating is present; however, further research is needed to entirely understand its role in improving population health. Further research should include longitudinal studies that assess the usual dietary intake over time more to confidently determine the relationship between dietary patterns with health and disease status. With an increasing body of scientific evidence, Canadian nutrition policies and practices are transitioning to the promotion of well-planned plant-based diets, such as the most recent iteration of the Canadian Food Guide. However, additional effective communication on the benefits of plant-based eating should continue. The promotion of plant-based diets should be incorporated within health promotion endeavours as plant-based diets can be a beneficial modifiable risk factor for many chronic conditions and support general health. Leadership and guidance should emphasize the beneficial connection between well-planned plant-based eating and health. The notion that not all plant-based diets are created equal needs to be effectively communicated to the Canadian population. The market for plant-based meat alternatives is rapidly growing and these novel products are in high demand due to their social popularity, easy preparation, and satisfying taste. However, knowledge translation on the healthfulness of these products is lacking; therefore, awareness that these highly processed plant-based meat alternatives may be high in sugar, fat, and/or salt and are detrimental to our health should be enhanced. As with any dietary pattern, special attention should be paid to ensure nutrients of concern are consumed in sufficient quantities when adopting a plant-based diet. Furthermore, prospective research studies should be conducted to better understand the relationships between plant-based diets, health, and the incidence of chronic conditions on a Canadian population. A well-planned plant-based diet can offer nutrient-dense diets which confer health benefits and may mitigate the risk of chronic conditions.

## Figures and Tables

**Table 1:** Sociodemographic, socioeconomic, and health characteristics of Canadians across three dietary patterns ^1^.

Characteristic	Diet (95% Confidence Interval)		
	Omnivore ^2^ (n = 14,776,319)	Semi-Plant-Based ^3^ (n = 2,354,977)	Strict Plant-Based ^4^ (n = 257,018)
Mean age, years	48.22 ± 0.29 (47.66–48.78)	49.82 ± 0.83 (48.20–51.45)	45.80 ± 2.02 (41.83–49.76)
Sex, % male	50.06 * ± 0.79 **(48.51–51.61)**	42.82 * ± 2.67 **(37.58–48.06)**	54.92 ± 8.25 (38.71–71.13)
Education, % post-secondary	77.09 ± 0.90 (75.31–78.85)	75.52 ± 2.21 (71.18–79.86)	86.35 ± 4.46 (77.58–95.12)
Marital status, % married	65.3 ± 1.14 (63.07–67.53)	64.16 ± 2.55 (59.16–69.18)	61.47 ± 8.40 (44.96–77.98)
Food secure ^5^, % yes	97.78 ± 0.60 (91.60–93.96)	93.44 ± 1.32 (90.84–96.04)	89.72 ± 4.46 (80.97–98.47)
Immigrant, % yes	26.66 * ± 1.21 **(24.28–29.04)**	31.03 ± 2.66 (25.80–36.25)	48.36 * ± 8.38 **(31.90–64.82)**
Recent immigrant ^6^, % yes	27.61 ± 2.05 (25.59–31.64)	31.83 ± 4.67 (22.66–41.00)	33.36 ± 11.75 (10.28–56.44)
Ethnicity, % white	74.87 ± 1.14 (72.64–77.11)	72.60 ± 2.69 (67.31–77.88)	- ^8^
Household income, % distribution decile 5 + 6	19.61 ± 0.83 (17.98–21.23)	21.83 ± 2.03 (17.83–25.82)	18.96 ± 6.02 (7.12–30.80)
Nutritional supplement consumption, % yes	42.26 ± 0.92 (44.46–48.05)	50.01 ± 2.17 (45.75–54.28)	44.01 ± 6.97 (43.29–69.69)
Physical activity, % met national recommendation ^7^	45.44 * ± 1.11 **(43.27–47.62)**	42.20 ± 2.60 (37.09–47.32)	26.74 * ± 6.52 **(13.94–39.55)**
Body mass index, % overweight	36.54 * ± 1.15 **(34.28–38.80)**	32.83 ± 2.41 (28.08–37.58)	20.64 * ± 6.08 **(8.69–32.59)**
Type 2 diabetes, % with diabetes	6.39 ± 0.45 (5.51–7.28)	6.48 ± 0.93 (4.65–8.30)	7.74 ± 3.68 (0.50–14.97)
Hypertension, % with hypertension	19.34 ± 0.82 (17.73–20.94)	17.63 ± 1.70 (14.30–20.97)	20.31 ± 5.47 (9.56–31.06)
Heart disease, % with heart disease	4.01 ± 0.38 (3.24–4.75)	5.19 ± 0.91 (3.41–6.97)	2.86 ± 1.61 (0.00–6.03)
Cancer, % with cancer	1.60 ± 0.20 (1.21–2.00)	1.02 ± 0.33 (0.38–1.66)	- ^7^
Osteoporosis, % with osteoporosis	4.18 ± 0.37 (3.45–4.91)	5.58 ± 0.94 (3.73–7.43)	1.31 ± 1.30 (0.00–3.87)
Total chronic condition, % with at least one condition	53.05 ± 1.48 (50.13–55.96)	52.07 ± 3.47 (45.26–58.87)	64.93 ± 8.22 (48.77–81.09)

Data source: 2015 Canadian Community Health Survey—Nutrition. * in bold Represents statistically significant difference as determined by 95% confidence interval approach. ^1^ All data are weighed and bootstrapped to obtained population level estimates. ^2^ Omnivore diet defined by the study population who reported consumption of any animal products. ^3^ Semi-plant-based diet defined by the study population who reported no consumption of animal products and reported consumption of animal by-products and at least one serving of fruits/vegetables. ^4^ Strict plant-based diet defined by the study population who reported no consumption of animal products or animal by-products but reported consumption of at least one serving of fruits/vegetables. ^5^ Food security was calculated using the 18-item US Household Food Security Survey Module. ^6^ Recent immigrant defined by less than 10 years in Canada. ^7^ National physical activity recommendation is 150 min of vigorous activity per week. ^8^ Low sample size (data were not released to adhere to Statistics Canada’s vetting guidelines).

**Table 2:** Nutrient-Rich Food index scores, as determined by the NRF index, of Canadians across three dietary patterns ^1^.

Nutrient-Rich Food Index Score ^2^	Diet (95% Confidence Interval)		
	Omnivore ^3^ (n = 14,776,319)	Semi-Plant-Based ^4^ (n = 2,354,977)	Strict Plant-Based ^5^ (n = 257,018)
% In Low NRF Index Score	35.41 * ± 1.15 **(33.15–37.67)**	25.70 * ± 2.43 **(20.92–30.48)**	35.70 ± 8.69 (18.62–52.77)
% In Medium NRF Index Score	32.77 ± 1.12 (30.56–43.97)	30.67 ± 2.36 (26.02–35.32)	35.22 ± 7.81 (19.87–50.56)
% In High NRF Index Score	31.82 * ± 1.04 **(29.79–33.86**)	43.63 * ± 2.63 **(38.47–48.79)**	29.09 ± 7.72 (13.91–44.26)
Mean NRF index score	517.13 * ± 2.86 (511.69–522.94)	544.03 * ± 6.79 (530.69–557.37)	521.50 ± 25.15 (472.10–750.91)

Data source: 2015 Canadian Community Health Survey—Nutrition. * in bold Represents statistically significant difference as determined by 95% confidence interval approach. ^1^ All data are weighed and bootstrapped to obtained population level estimates. ^2^ Nutrient-Rich Food index score categories as based on tertiles. ^3^ Omnivore diet defined by the study population who reported consumption of any animal products. ^4^ Semi-plant-based diet defined by the study population who reported no consumption of animal products and reported consumption of animal by-products and at least one serving of fruits/vegetables. ^5^ Strict plant-based diet defined by the study population who reported no consumption of animal products or animal by-products but reported consumption of at least one serving of fruits/vegetables.

**Table 3:** Mean micronutrient intakes of Canadians across three dietary patterns ^1^.

Nutrient (Unit)	Diet (95% Confidence Interval)		
	Omnivore ^2^ (n = 14,776,319)	Semi-Plant-Based ^3^ (n = 2,354,977)	Strict Plant-Based ^4^ (n = 257,018)
Vitamin A (mcg)	**666.42 ^a^ ± 14.66** **(637.62–695.22)**	**671.42 ^a,b^ ± 25.99** **(620.36–722.49)**	**449.50 ^b^ ± 90.98** **(270.75–628.25)**
Vitamin B6 (mg)	**1.78 ^a^ ± 0.02** **(1.74–1.82)**	**1.53 ^b^ ± 0.06** **(1.41–1.65)**	**1.42 ^b^ ± 0.13** **(1.17–1.67)**
Vitamin B12 (mcg)	**4.45 ^a^ ± 0.09** **(4.27–4.63)**	**2.93 ^b^ ± 0.16** **(2.61–3.25)**	**0.65 ^c^ ± 0.15** **(0.35–0.94)**
Vitamin C (mcg)	101.76 ^a^ ± 2.28 (97.27–106.25)	10.3.67 ^a^ ± 4.86 (94.12–113.22)	119.98 ^a^ ± 19.72 (81.24–158.71)
Vitamin D (mcg)	**4.92 ^a^ ± 0.12** **(4.69–5.16)**	**4.41 ^a^ ± 0.18** **(4.05–4.77)**	**1.30 ^b^ ± 0.33** **(0.66–1.95)**
Calcium (mg)	**799.59 ^a^ ± 12.23** **(775.56–823.61)**	**872.92 ^a^ ± 28.11** **(817.70–928.14)**	**418.90 ^b^ ± 53.41** **(313.97–523.84)**
Cholesterol (mg)	**291.09 ^a^ ± 6.65** **(278.02–304.17)**	**235.03 ^b^ ± 13.66** **(208.19–261.88)**	**47.36 ^c^ ± 6.86** **(33.87–60.84)**
MUFAs ^5^ (g)	**27.32 ^a^ ± 0.38** **(26.57–28.06)**	**25.64 ^a^ ± 0.85** **(23.98–27.31)**	**16.72 ^b^ ± 1.76** **(13.26–20.19)**
PUFAs ^6^ (g)	15.17 ^a^ ± 0.23 (14.72–15.62)	15.07 ^a^ ± 0.56 13.97–16.16)	14.23 ^a^ ± 1.60 (11.09–17.38)
Saturated Fatty Acids (g)	**23.75 ^a^ ± 0.35 ^a^** **(23.06–24.44)**	**22.78 ^a^ ± 0.82** **(21.17–24.40)**	**11.78 ^b^ ± 1.28** **(9.07–14.49)**
Dietary Fibre (g)	**17.27 ^a^ ± 0.21** **(16.85–17.69)**	**20.36 ^b^ ± 0.61** **(19.5 1- 21.56)**	20.42 ^a,b^ ± 3.00 (14.51–26.32)
Folate (mcg)	**215.69 ^a^ ± 2.96** **(209.88–221.50)**	**245.86 ^b^ ± 8.85** **(228.47–263.26)**	241.74 ^a,b^ ± 24.29 (194.28–289.47)
Iron (mg)	**12.86 ^a^ ± 0.13** **(12.06–13.13)**	12.16 ^a,b^ ± 0.36 (11.46–12.86)	**10.48 ^b^ ± 1.07** **(8.37–12.59)**
Magnesium (mg)	315.60 ^a^ ± 3.06 (309.58–321.63)	323.20 ^a^ ± 9.74 9304.05–324.34)	285.33 ^a^ ± 32.03 (222.40–348.25)
Niacin (mg)	**42.37 ^a^ ± 0.45** **(41.48–43.25)**	**30.26 ^b^ ± 1.00** **(28.30–32.23)**	**22.13 ^c^ ± 1.61** **(18.97–25.29)**
Phosphorus (mg)	**1336.73 ^a^ ± 13.61** **(1309.99–1363.47)**	**1224.64 ^b^ ± 39.25** **(1127.52–1301.76)**	**851.39 ^c^ ± 87.80** **(678.89–1023.90)**
Potassium (mg)	2794.49 ^a^ ± 25.68 (2744.04–2844.94)	2674.41 ^a^ ±70.59 (2535.72–2813.10)	2317.59 ^a^ ± 238.63 (1848.74–2786.44)
Riboflavin (mg)	**1.99 ^a^ ± 0.02** **(1.95–2.03)**	**1.92 ^a^ ± 0.06** **(1.81–2.04)**	**1.05 ^b^ ± 0.09** **(0.86–1.23)**
Sodium (mg)	**2845.16 ^a^ ± 35.63** **(2775.15–2915.16)**	**2630.72 ^a^ ± 88.83** **(2456.18–2805.25)**	**1695.07 ^b^ ± 175.46** **(1250.33–2039.81)**
Thiamin (mg)	**1.64 ^a^ ± 0.02** **(1.60–1.68)**	1.62 ^a, b^ ± 0.05 (1.51–1.73)	**1.27 ^b^ ± 0.15** **(0.97–1.57)**
Zinc (mg)	**11.31 ^a^ ± 0.14** **(11.03–11.59)**	**8.18 ^b^ ± 0.28** **(7.63–8.73)**	**6.03 ^c^ ± 0.66** **(4.73–7.33)**

Data source: 2015 Canadian Community Health Survey—Nutrition. ^a,b,c^ in bold Represents statistically significant difference, as determined by 95% confidence interval approach, between indicated dietary groups, such that means with different superscripts are statistically different. ^1^ All data are weighed and bootstrapped to obtained population level estimates. ^2^ Omnivore diet defined by the study population who reported consumption of any animal products. ^3^ Semi-plant-based diet defined by the study population who reported no consumption of animal products and reported consumption of animal by-products and at least one serving of fruits/vegetables. ^4^ Strict plant-based diet defined by the study population who reported no consumption of animal products or animal by-products but reported consumption of at least one serving of fruits/vegetables. ^5^ Mono-unsaturated fatty acids. ^6^ Poly-unsaturated fatty acids.

**Table 4:** Mean macronutrient intakes of Canadians across three dietary patterns ^1^.

Nutrient (Unit)	Diet (95% Confidence Interval)		
	Omnivore ^2^ (n = 14,776,319)	Semi-Plant-Based ^3^ (n = 2,354,977)	Strict Plant-Based ^4^ (n = 257,018)
Total Carbohydrate (g)	227.58 ^a^ ± 2.18 (223.30–231.86)	238.98 ^a^ ± 5.90 (227.39–250.58)	215.03 ^a^ ± 23.10 169.64–260.41)
Total Fat (g)	**72.79 ^a^ ± 0.94** **(70.94–74.63)**	**69.80 ^a^ ± 2.21** **(65.47–74.13)**	**46.84 ^b^ ± 3.66** **(39.65–54.03)**
Total Protein (g)	**85.95 ^a^ ± 0.90** **(84.17–87.72)**	**61.05 ^b^ ± 1.84** **(57.44–64.66)**	**40.73 ^c^ ± 3.09** **(34.66–46.80)**
Total Sugar (g)	88.67 ^a^ ± 1.17 (86.37–90.97)	93.41 ^a^ ± 3.07 (87.38–99.43)	77.88 ^a^ ± 10.58 (57.10–98.66)
Total Energy (kcal)	**1952.98 ^a^ ± 18.07** **(1917.48–1988.48)**	**1849.29 ^a^ ± 44.71** **(1761.45–1937.12)**	**1421.57 ^b^ ± 121.12** **(1183.61–1659.54)**

Data source: 2015 Canadian Community Health Survey—Nutrition. ^a,b,c^ in bold Represents statistically significant difference, as determined by 95% confidence interval approach, between indicated dietary groups, such that means with different superscripts are statistically different. ^1^ All data are weighed and bootstrapped to obtained population level estimates. ^2^ Omnivore diet defined by the study population who reported consumption of any animal products. ^3^ Semi-plant-based diet defined by the study population who reported no consumption of animal products and reported consumption of animal by-products and at least one serving of fruits/vegetables. ^4^ Strict plant-based diet defined by the study population who reported no consumption of animal products or animal by-products but reported consumption of at least one serving of fruits/vegetables.

## Data Availability

Restrictions apply to the availability of these data. Data were obtained from Statistics Canada and are available at local Research Data Centres with the permission of Statistics Canada.
